# *Bacillus velezensis* TSA32-1 as a Promising Agent for Biocontrol of Plant Pathogenic Fungi

**DOI:** 10.3390/jof8101053

**Published:** 2022-10-08

**Authors:** Jung-Ae Kim, Jeong-Sup Song, Pyoung Il Kim, Dae-Hyuk Kim, Yangseon Kim

**Affiliations:** 1 Department of Research and Development, Center for Industrialization of Agricultural and Livestock Microorganisms, Jeongeup-si 56212, Korea; 2 Department of Bioactive Material Science, Jeonbuk National University, Jeonju 54896, Korea; 3 Department of Molecular Biology, Institute for Molecular Biology and Genetics, Jeonbuk National University, Jeonju 54896, Korea

**Keywords:** antifungal activity, *Bacillus velezensis*, biological control, phytopathogen, genome-sequence analysis

## Abstract

The use of synthetic fungicides has caused major problems such as soil and water pollution and negatively affects non-target species. Microbial biocontrol agents are needed for crop disease management to reduce agrochemical use. *Bacillus* and related genera produce secondary metabolites with agricultural applications, such as the pathogen-control agent *Bacillus velezensis.* We isolated *B. velezensis* TSA32-1 from soil and identified its characteristics by sequencing its 16S rRNA. *B. velezensis* TSA32-1 showed enzyme activity and antimicrobial effects against phytopathogenic fungi by inhibiting the growth of *Fusarium graminearum*, *F. fujikuroi*, *Alternatia alternate*, and *Diaporthe actinidiae.* Additionally, *B. velezensis* TSA32-1 protected diseases in corn and pepper seeds caused by *F. graminearum* and *Pythium ultimum*. The complete genome of *B. velezensis* TSA32-1 was 4.05 Mb with a G+C content of 46.3 mol % and possessed the bacillaene biosynthesis cluster, a polyketide that inhibits protein biosynthesis. We also detected a surfactin synthesis cluster, known as non-ribosomal peptide synthetases, which biosynthesizes the antibacterial substance lipopeptide. Surfactin, and fengycin family compounds, secondary metabolites known as key factors in biological control, also detected *B. velezensis* TSA32-1 which shows potential as a biocontrol agent for controlling plant pathogens in agriculture.

## 1. Introduction

In agriculture, biotic stressors such as pathogens and pests cause 20–30% of global crop yield losses [1]. Particularly, soil-borne fungal infections of important grain crops such as corn, rice, and pepper cause large economic losses by reducing yields [2]. Chemicals for protecting crops and promoting growth such as pesticides, herbicides, and fertilizers are widely used in the agricultural sector to ensure sufficient and consistent yields [3]. However, many of these chemicals lead to soil and water pollution and pesticide residues in agricultural products, negatively affecting human health and the environment [4]. Thus, organic products as safe and eco-friendly control technologies, such as biological agents, are needed for disease prevention and management in agriculture [5]. The use of microorganisms as biological agents that can control disease-causing plant pathogens in an environmentally sound manner has been widely reported [6,7].

The biocontrol abilities of these microbial agents are typically related to the secondary metabolite profile of the microorganism. Antagonist microorganisms produce several secondary metabolites that can control various phytopathogens. Representatively, some strains of *Bacillus velezensis*, *B. siamensis*, and *B. subtilis* have been proposed as plant pathogen control agents because they produce numerous antimicrobial compounds, such as surfactin, iturin, bacilysin, fengycin, macrolactin, bacillaene, and difficidin [8,9]. In addition, the *Bacillus* species have the advantage of growing rapidly under simple trophic conditions and produce endospores that make cells more resistant to environmental stress [10]. *B. subtilis* SG6 showed antagonistic action against the *F. graminearum* pathogen, and extraction of secondary metabolites of surfactin and fengycin has been reported [11]. *B. subtilis* BS8B-1 shows antagonistic effects to the *Pythium* pathogen under greenhouse conditions; genetic approaches and chemical analysis were used to evaluate the mechanism of volatile substances and antibacterial substances against pathogenic fungi [12]. *B. inaquosorum* KR2-7 has been proposed as an effective biological control agent against *F. oxysporum* under greenhouse conditions. Genome mining was performed to explain these antifungal effects, and fengycin, surfactin, and iturin, among others, were detected as secondary metabolites [13]. Li et al. (2021) also reported performing genome mining and mass spectrometry analysis to analyze the various secondary metabolites serving as biological agents of *B. velezensis* W1 to seek antimicrobial active ingredients [14].

In this study, we aimed to investigate isolates, and identify functional characteristics of *B. velezensis* TSA32-1 for developing biological control agents against *Fusarium* and *Pythium*, pathogens of important crops such as corn and pepper. The antifungal effect of TSA32-1 was detected against soil-borne pathogens by treating corn and pepper seeds with *B. velezensis* TSA32-1. We provide an understanding of the mechanism of action of *B. velezensis* TSA32-1 biological agents by detecting secondary metabolites such as surfactin and plipastatin in the *B. velezensis* TSA32-1 culture medium through chemical analysis. Based on the diverse and effective properties of TSA32-1 for its antifungal action, we propose TSA32-1 as a beneficial biocontrol agent for the eco-friendly agricultural industry.

## 2. Materials and Methods

### 2.1. Isolation and Identification of B. velezensis TSA 32-1

*B. velezensis* TSA32-1 was collected from soil (Muan-gun, Korea) and isolated using the serial dilution method [15]. Soil samples (1 g) were processed by crushing, suspended in physiological saline (9 mL), and homogenized. For enumeration, a 10-fold dilution series of each homogenate was prepared in sterile saline solution, and 0.1 mL of this sample was spread on tropical soy agar plates, which were incubated at 30 °C for 24 h. As reference stains, *B. subtilis* KCTC3135 and *B. velezensis* KCTC13012 were obtained from the Korean Collection for Type Cultures (Jeollabuk-do, Korea).

### 2.2. Phylogenetic and Statistical Analysis

The isolated strains were sequenced and identified using 16S rRNA sequencing. Phylogenetic analysis was conducted using the maximum likelihood method, with the numbers at the branch points indicating the bootstrapping values, using Molecular Evolutionary Genetic Analysis software, version 7.0 [16]. The data were determined as the average value of three independent biological repetitions. Standard deviations represent variation among replicates. One-way analysis of variance (Tukey’s multiple comparison test) was performed using GraphPad Prism 5 software (GraphPad, Inc., La Jolla, CA, USA) to detect statistically significant differences (*p* < 0.05) between treatments [17].

### 2.3. Enzymatic Activity Analysis

#### 2.3.1. Cellulase Activity

Cellulase activity was evaluated through a Cellulase Activity Assay Kit (ab189817; Abcam, Cambridge, UK) according to the manufacturer’s instructions. Cellulase activity was measured as the fluorescence at Ex/Em = 550/595 nm (peak Ex/Em = 571/585 nm) in a microplate reader in kinetic mode for 120min at 25 °C. The sample concentration was calculated using a standard curve.

#### 2.3.2. Lysozyme Activity

Microbial resistance to lysosomal hydrolytic enzymes was evaluated using a Lysozyme Assay Kit (E-22013; Thermo Fisher Scientific, Waltham, MA, USA) according to the manufacturer’s instructions with a fluorescent-based microplate reader. Lysosome activity was quantified by measuring the fluorescence intensity at 450 nm.

#### 2.3.3. Protease Activity

Protease activity was evaluated using a Protease Activity Assay Kit (ab111750, Abcam) according to the manufacturer’s instructions. Fluorescence was measured at Ex/Em = 485/530 nm and then re-measured after 30 min. Protease activity was determined according to standard values and Equation (1). In Equation (1), B is the amount of fluorescein isothiocyanate (nmol) from the standard curve, T1 is the time (min) of the first reading (R1), T2 is the time (min) of the second reading (R2), and V is the pretreated sample volume (mL) added to the reaction well.

(1) $$\mathrm{Protease}\;\mathrm{activity}\left(\mathrm{mU}/\mathrm{mL}\right)=\frac{B\;\times \;\mathrm{dilution}\;\mathrm{factor}}{\left(T2-T1\right)\;\times \;V}$$

### 2.4. Antifungal Analysis

The antifungal ability of TSA32-1 was evaluated using the disk diffusion method as previously described [14] with slight modifications against several phytopathogenic fungi from the Korean Agricultural Culture Collection (KACC, Jeollabuk-do, Korea). Briefly, the fungal pathogen was cultured on a potato dextrose agar plate for 7 days. A pathogenic agar block with a diameter of 8 mm was prepared and placed in the center of the plate. A diffusion disk with a diameter of 8 mm was overlaid on the agar, and 20 µL of 1 × 10^6^ colony forming units/mL of the culture suspension was distributed on the right disk; 20 µL of distilled water was added to the left disk as a control. After culturing at 25 °C for 3–5 days, the antifungal activity of TSA32-1 was assessed by determining the radial mycelial growth of the fungal pathogen. All experiments were performed in triplicate.

### 2.5. Pathogen Inoculum Production and Disease Evaluation

#### 2.5.1. Preparation of Bacillus Strains

The *Bacillus* strains were prepared at 1 × 10^6^ colony-forming units/mL or higher by incubation overnight in a 30 °C shaking incubator at 150 rpm in a tryptic soy broth medium. The sample was centrifuged at 4 °C and 10,000 rpm and the pellet was collected. The pellet was resuspended in 250 ppm of Tween 20.

#### 2.5.2. Preparation of Seeds

Corn seeds (*Zea mays* L.) and pepper seeds (*Capsicum annuum* L.) were disinfected by immersion in 100% ethanol for 1 min, rinsed three times with water, immersed in 1% sodium hypochlorite for 30 min, and rinsed three times with water [18]. The seeds were dehydrated overnight in a hood.

#### 2.5.3. Preparation of Pathogenic Fungus

*F. graminearum* KACC41044 was incubated in potato dextrose agar medium at 25 °C for 7 days and then cut into agar blocks. The agar blocks were inoculated with 15 g carboxymethyl cellulose, 1 g NH_4_NO_3_, 1 g KH_2_PO_4_, 0.5 g MgSO_4_∙7H_2_O, 1 g yeast extract, and 1 L distilled water in a medium and incubated at 25 °C in a shaking incubator at 200 rpm for 5 days. After filtering the culture solution through a sterile Miracloth to filter mycelium, the filtrate was centrifuged at 4 °C at 10,000 rpm. The pellet was collected and mixed with 250 ppm of Tween 20, and microconidia were counted using a hemocytometer [19].

*Pythium ultimum* KACC40705 was prepared by culturing in corn-meal agar medium at 25 °C for 2 days. Several agar blocks of *P. ultimum* KACC40705 were cut and inoculated onto sterilized rice, with mixing once daily and incubation in the dark at room temperature for 2 weeks [20]. The pathogens were filtered through a sterile Miracloth and resuspended in 250 ppm Tween 20 [21].

#### 2.5.4. Seed Germination Rate of Treatments

The seed germination rate of corn and pepper was investigated following coating with the TSA32-1 culture. The seed germination rate was estimated as a percentage based on the average percentage of the control seed germination value, which was considered as 100. For pathogen inoculation, corn seeds soaked for 30 min in the prepared *Bacillus* immersion solution at room temperature were sprayed with the microconidia of *F. graminearum* KACC41044 pathogen at 1 × 10^6^ microconidia/mL. As a control, untreated corn seeds were placed on filter paper and moistened with distilled water. The pathogen-treated corn seeds were placed on filter paper and directly sprayed with the conidia suspension. The seed germination rate was measured after culturing for 1 week in the dark at 25 °C while maintaining moist conditions.

Pepper seeds, as in the corn seed experiments, were induced to germinate by placing the seeds on a filter paper and supplying moisture as described by Shang et al. (1998) with some modifications. *P. ultimum* KACC40705 was used as the pathogen [22].

#### 2.5.5. Disease Suppression Effect of Seed Treatments

The ability of TSA32-1 to suppress the disease caused by *F. graminearum* and *P. ulimum* on corn and pepper was evaluated in pot tests with autoclaved soil [20,23]. The corn and pepper seeds, either coated or uncoated with TSA32-1 were sowed into the sterilized soil (50 g) and placed in a plastic pot with a diameter of approximately 60 mm and a height of 65 mm. Three seeds were sown in each pot, and the experiment was performed with 10 pots for each configuration. *F. graminearum* KACC41044 and *P. ultimum* KACC40705 were inoculated into the soil to induce disease. The conidia suspension of the *F. graminearum* pathogen was prepared at a concentration of 1 × 10^6^ microconidia/mL and 1 mL was inoculated into each pot of soil. The disease was induced by adding 5 g of the *P. ultimum* pathogen prepared by infection with rice, to each pot of 50 g of sterile soil [20]. The tests were conducted in a humid greenhouse at 20–25 °C with 12-h photoperiods for 10 days. All tests were performed in triplicate.

#### 2.5.6. DNA Extraction and Whole-Genome Sequencing

Genomic DNA was extracted using a DNeasy UltraClean microbial kit (Qiagen, Hilden, Germany) according to the manufacturer’s instructions. Whole-genome sequencing was performed using the Pacific Biosciences (PacBio) RS II Single-Molecule Real-Time (SMRT) platform with a 20 kb SMRTbell™ template library at ChunLab. The reads were assembled using PacBio SMAR Analysis 2.3.0 and an Illumina Hi-Seq system (San Diego, CA, USA) at Macrogen (Seoul, Korea).

#### 2.5.7. Annotation

The single-molecule real-time sequencing reads were assembled de novo using the hierarchical genome assembly process workflow (HGAP 3.0) in PacBio’s SMRT portal with subreads from PacBio. Paired-end reads from Illumina Hi-Seq 2500 were mapped to the assembled contigs to improve the accuracy of the genome sequences [24]. The sequences were annotated using the combined results of the automated National Center for Biotechnology Information Prokaryotic Genomes Annotation Pipeline and Rapid Annotations Subsystems Technology prokaryotic genome annotation server (http://rast.nmpdr.org/, version 2.0, accessed on 1 August 2022) [25]. The coding genes were predicted according to clusters of orthologous groups using the WebMGA online tool [26].

### 2.6. Antifungal Metabolite Extraction and Analysis

#### 2.6.1. Preparation of Culture Extract

The culture medium used to grow TSA 32-1 and identify secondary metabolites has been described by Kim et al. (2010) [27]. TSA 32-1 was cultured in 1 L tryptic soy broth medium at 30 °C for 48 h and centrifuged to obtain the cell-free extract. HCl (1 N, 40 mL) was added to 1 L of the extract, and the mixture was protonated at 4 °C for 24 h to adjust the pH to 3.1. The mixture was centrifuged, and the pellet was dissolved in methanol and concentrated on a rotary evaporator (R-100, Büchi, Flawil, Switzerland). After filtration through a 0.2 μm filter, liquid chromatography–quantitative time-of-flight (LC–QTOF) analysis was performed. The 24-h culture medium of TSA32-1 was used for bacillaene analysis.

#### 2.6.2. Ultra-High Performance (UP) LC-QTOF-Mass Spectrometry (MS) Analysis of Metabolites

The chemical profile of the metabolites produced by TSA32-1 was analyzed using a Waters Acquity UPLC I-Class PLUS equipped with a Waters ACQUITY UPLC BEH C18 column (2.1 × 100 mm, 1.7 µm) (Milford, MA, USA), maintained at an isothermal temperature of 65 °C. A binary pump delivered the mobile phase at the flow rate of 0.4 mL/min under gradient elution using two mobile phases, LC-MS–grade water + 0.1% *v*/*v* formic acid (solvent A) and LC-MS–grade acetonitrile + 0.1% *v*/*v* formic acid (solvent B). The auto-sampler was set to an injection volume of 1 µL. MS analysis was performed using a Waters Xevo-G2-XS QTOF LC-MS equipped with an electrospray ionization source. An analysis was conducted in positive-ion mode at a mass range of 100–1600 Da. The source temperature and capillary voltage were set to 150 °C and 3.0 kV, respectively. Ar was used as the collision gas. Standard solutions of surfactin (Santa Cruz Biotechnology, Dallas, TX, USA) and plipastatin (Sigma, St. Louis, MI, USA) were used.

## 3. Results

### 3.1. Identification of Bacterial Strains

**Figure 1:**
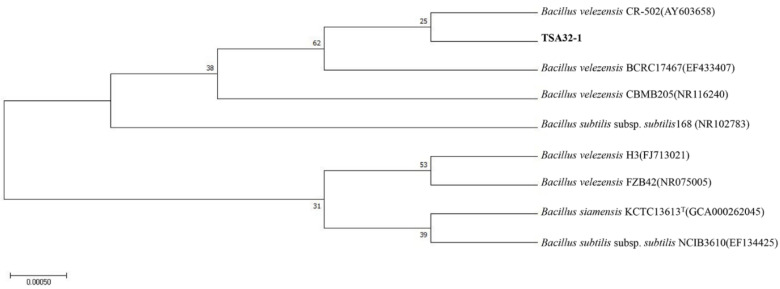
16S rRNA phylogenetic analysis of selected strain. Phylogenetic tree based on maximum likelihood method of 16S rRNA gene sequences of TSA32-1. The tree was created using the maximum likelihood method and numbers at the branch points are bootstrap values (based on 500 samplings expressed in percentages).

We isolated TSA32-1 from soil and identified the species based on the 16S rRNA sequences. The genomic DNA sequences of the strains were searched against the available gene sequences in the GenBank database. Strain TSA32-1 is 99% homologous to *B. velezensis* (AY603658). The phylogenic relationship between the bacterial strains was established based on the maximum likelihood of evolutionary distances of the 16S rRNA sequences, as shown in Figure 1.

### 3.2. Plant Disease Biocontrol Activity

#### 3.2.1. Enzyme Activity of Isolated Strains

**Figure 2:**
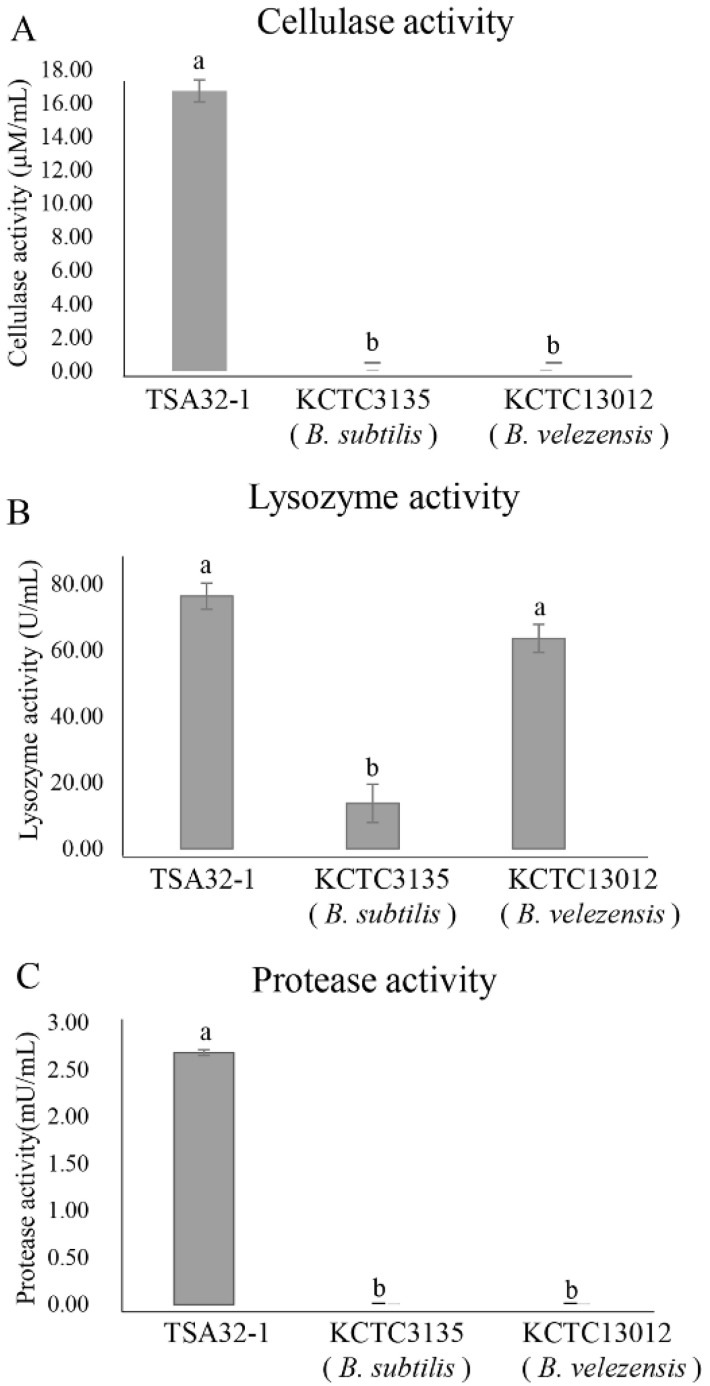
Enzyme activity of *Bacillus* spp. (**A**) Cellulase enzymes on the cell wall of TSA32-1. (**B**) Lysosomal hydrolytic enzymes on the cell wall of TSA32-1. (**C**) Protease activity of TSA32-1. Data are expressed as the means ± SD. Significant differences are marked with letters (*p* < 0.05). Reference strains *Bacillus subtilis* KCTC3135, *B. velezensis* KCTC13012 were tested as controls.

The antifungal effects of cellulolytic, lysosomes, and proteolytic enzymes were evaluated, and TSA32-1 substantially affected the cell wall-related three enzyme treatment as shown in Figure 2. The measured cellulase activity of TSA32-1 was 16.75 μM/mL, which demonstrated marked resistance to cellulose activity as displayed with higher activity than the type strains of *B. velezensis* KCTC 13012 and *B. subtilis* KCTC3135 (Figure 2A). The measured lysozyme activity of TSA32-1 was 76.46 U/mL, *B. velezensis* KCTC 13012 was 63.65 U/mL, and *B. subtilis* KCTC3135 was 13.73 U/mL, respectively, indicating superior or equivalent lysosomal activity to the type strain (Figure 2B). Similarly, the measured protease activity of TSA32-1 was 2.68 mU/mL, which showed higher activity than the type strain (Figure 2C). TSA32-1 was superior to the type strain in three enzyme activities, cellulase, lysozyme, and protease, which are enzymes that help inhibit fungal growth. These results indicate that increased production and activity of cell wall degrading enzymes of TSA32-1 are associated with decreased invasiveness and pathogenicity.

#### 3.2.2. In Vitro Antifungal Effects against Phytopathogenic Fungi

**Figure 3:**
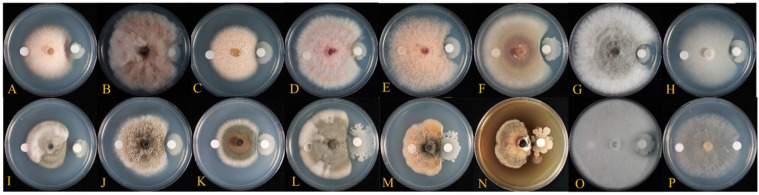
Antifungal activity of test strains against the indicator strains. Inhibition effect of mycelial growth of *Fusarium fujikuroi* KACC 44002 (**A**), *F. graminearum* KACC 41040 (**B**), *F. graminearum* KACC 46434 (**C**), *F. graminearum* KACC 41044 (**D**), *F. oxysporum* KACC 41088 (**E**), *F. oxysporum f.* sp. *Raicis-lycopersici* O1090 (**F**), *Botryosphaeria dothidea* KACC 45481 (**G**), *Botrytis cinerea* KACC 47009 (**H**), *Alternaria alternata* KACC 45440 (**I**), *Diaporthe actinidiae* SN18 (**J**), *Glomerella cingulate* 06-KN-1 (**K**), *Colletotrichum nymphaea* GNC1 (**L**), *Colletotrichum acutatum* KACC 40042 (**M**), *Cylindrocarpon destructans* KACC 41077 (**N**), *Pythium ultimum* KACC40705 (**O**), and *Rhizoctonia solani* AG-1 KACC 40101 (**P**) by *B. velezensis* TSA32-1.

The antagonistic activity assay showed that TSA32-1 inhibited the filamentous growth of 16 fungal phytopathogens (Figure 3). TSA32-1 strongly inhibited mycelial growth of *Alternaria alternate* KACC45440 (brown ring spot of kiwi fruit), *Collectrichum acutatum* KACC40042 (anthracnose of pepper), *C. lymphaea* GNC1 (anthracnose of kiwi berry), *Cylindrocarpon destructans* KACC41077 (brown colony of ginseng), *Diaporthe actinidiae* SN18 (stem-end rot of kiwi fruit), and *F. oxysporum f.* sp. *Raicis-lycopersici* O1090 (root rot of tomato) compared to the control. TSA32-1 also inhibited the mycelial growth of *Botryosphaeria dothidea* KACC45481 (circular growth of crab apple), *Botrytis cinereal* KACC47009 (gray mold of grape), *F. graminearum* KACC41044 (head blight of corn), *F. graminearum* KACC46434 (scab of rice), *F. oxysporum* KACC41088 (root rot of orchids), *F. fugikuroi* KACC44002 (bakanae disease of rice), *Glomerella cingulate* 06-KN-1 (anthracnose of ki-wifruit), and *P. ultimum* KACC40705 (damping-off of cucumber).

#### 3.2.3. Plant Protection Effects of Seed Treatments with TSA32-1

**Figure 4:**
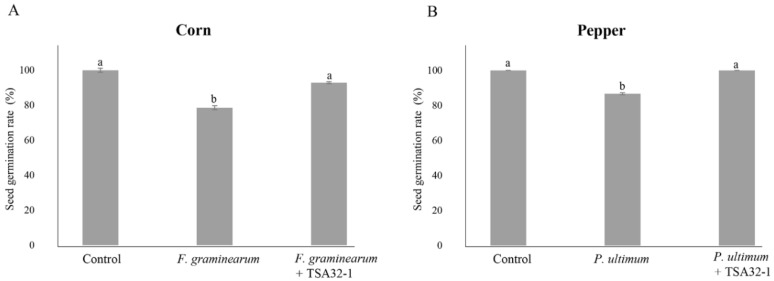
The seed germination rate of seed treatments. (**A**) Treatments of the prototype TSA32-1 for head blight disease in corn caused by *Fusarium graminearum* KACC41044. (**B**) Treatments of the prototype TSA32-1 for damping-off disease in pepper caused by *Pythium ultimum* KACC40705. Data are expressed as the means ± SD. Significant differences are marked with letters (*p* < 0.05). Control, untreated check; fungal pathogen, infected pathogen; fungal pathogen + TSA32-1, pathogen inoculation after seed dipping.

The germination rate of seeds infected with the phytopathogenic fungus *F. graminearum* KACC41044 on corn seeds was 78.6%, and seeds coated with TSA32-1 showed a germination rate of 92.9% even after pathogen infection (Figure 4A). When the seeds were treated with TSA32-1, the germination rate was higher than that of corn seeds infected with the pathogen alone. In addition, pepper seeds coated with TSA32-1 showed a germination rate of 100% even after pathogen infection, whereas pepper seeds infected with *P. ultimum* KACC40705 showed a germination rate of 88.7% (Figure 4B). These results suggest that TSA32-1 antagonizes *F. graminearum* and *P. ultimum* and positively affects the seed germination rate, which directly affects crop growth.

**Figure 5:**
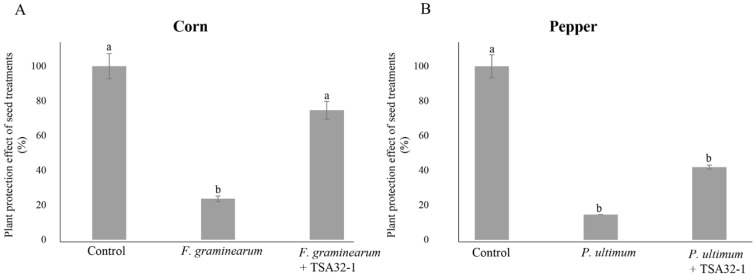
Plant protection effect of seed treatments. (**A**) Biocontrol agent effect of treatments of the prototype TSA32-1 for head blight disease in corn caused by *Fusarium graminearum*. (**B**) Biocontrol agent effect of treatments of the prototype TSA32-1 for damping-off disease in pepper caused by *Pythium ultimum*. Data are expressed as the means ± SD. Significant differences are marked with letters (*p* < 0.05). Control, untreated check; fungal pathogen, infected pathogen; fungal pathogen + TSA32-1, pathogen inoculation after seed dipping.

The control value was set as 100%, and the data were expressed as percentages relative to this value. The germination rate of TSA32-1-treated and pathogen-infected corn seeds in the soil was 74.6%, whereas the germination rate of corn sprouts was 23.6% when inoculated with the pathogen *F. graminearum* KACC41044 alone (Figure 5A). Similar in vitro seed germination rates were observed, with TSA32-1 showing antifungal action against *F. graminearum* pathogens and enabling seedling growth. The germination rate of pepper seeds infected with the *P. ultimum* KACC40705 pathogen alone was 14.5%, whereas pathogen-infected pepper seeds pre-coated with TSA32-1 showed a germination rate of 41.9% (Figure 5B). The results of measuring sprout germination rate by infecting red pepper seeds treated with TSA32-1 with pathogens tend to show higher values than those treated with pathogens alone.

### 3.3. Genome Feature of B. velezensis TSA32-1

**Figure 6:**
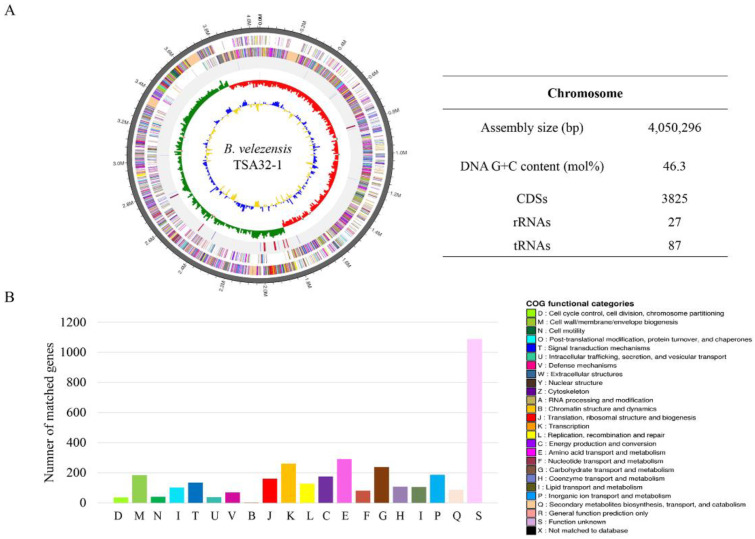
Genome features of *Bacillus velezensis* TSA32-1. (**A**) Circular genome maps of *B. velezensis* TSA32-1 chromosome. Circles from the outside to the center denote rRNA and tRNA gene, reverse strand coding sequence, forward strand coding sequence, GC skew, and GC content. (**B**) Genome number of clusters of orthologous groups category.

The complete genome of TSA32-1 was composed of a 4,050,296 base pair circular chromosome with a 46.3% G+C content. The genome contains 3825 protein-coding sequences, 27 rRNAs, and 87 tRNAs (Figure 6). Among the coding sequences, 3512 genes were classified into 20 clusters of orthologous groups’ functional categories (Figure 6B). Many genes were classified into functional categories for amino acid transport and metabolism (*n* = 291), transcription (*n* = 261), carbohydrate transport and metabolism (*n* = 238), inorganic ion transport and metabolism (*n* = 186), cell wall/membrane/envelope biogenesis (*n* = 184), energy production and conversion (*n* = 175), translation (*n* = 161), signal transduction mechanisms (*n* = 134), and recombination and repair (*n* = 127). Interestingly, 15.1% of genes were involved in amino acid transport and metabolism, carbohydrate transport, and metabolism.

The gene clusters related to the antibiotic activity identified in the genome of TSA 32-1 and secondary metabolites synthesized during gene expression are summarized in Table 1. Interestingly, TSA32-1 possesses five non-ribosomal peptide synthetases (NRPS) metabolite clusters within its genome of a large size of over 20 kb, which are conserved in all *B. velezensis* members. This group of three NRPS clusters comprises surfactin, plipastatin, and bacilysin and two polyketide synthetase-NRPS gene clusters, encoding the anti-bacterial polyketide bacillaene and antifungal polyketide mycosubtilin (Table 1). The whole genome sequences of the isolates have been deposited in GenBank under Bioproject number PRJNA602208 for *B. velezensis* TSA32-1.

### 3.4. Antifungal Metabolite of B. velezensis TSA32-1

**Figure 7:**
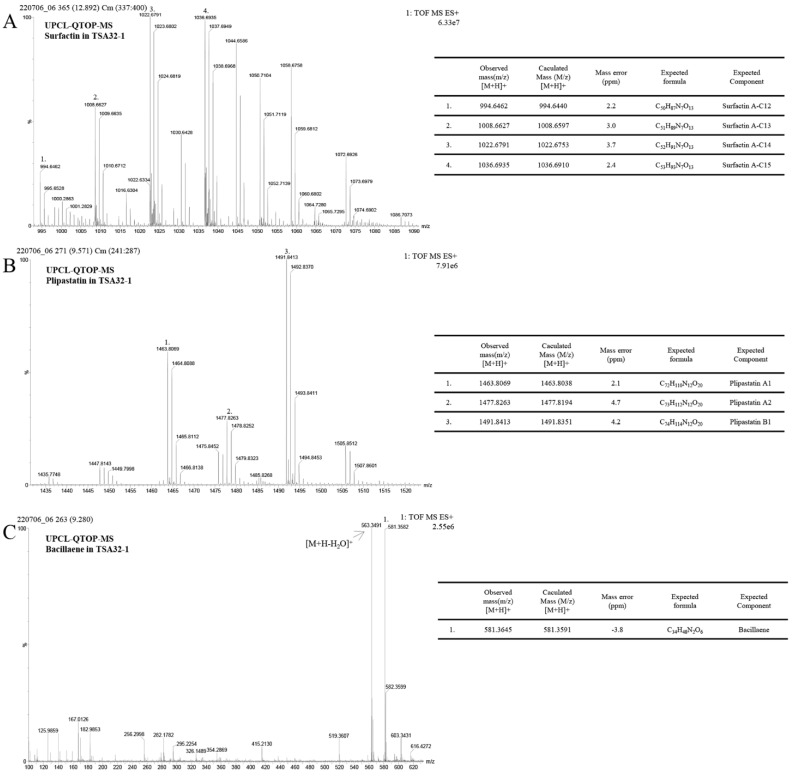
Identification of signaling metabolites in *Bacillus velezensis* TSA32-1. Extracts were fractioned using UPLC-QTOF-MS. (**A**) Mass spectrum of surfactin, (**B**) plipastatin, (**C**) bacillaene compounds in TSA32-1 culture extract.

Lipopeptides produced by TSA32-1 were identified using UPLC–QTOF–MS analysis. Standard solutions of surfactin and plipastatin generated four major peaks. Similarly, crude surfactin gained from the extracts of TSA32-1 culture was further purified via preparative chromatography with a solvent containing methanol, and the partial peak was resolved into four fractions. Each fraction revealed that the sample contained quasi molecular ions at mass-to-charge ratios (*m*/*z*) of 994.6462, 1008.6629, 1022.6791, and 1036.6935 ([M + H]^+^) indicating surfactin (Figure 7A). Another Q-TOF-MS analysis revealed three fractions for plipastatin with *m*/*z* of 1463.8069, 1477.8263, and 1491.8413 representing plipastatin A–B, respectively (Figure 7B). There were also well-resolved peaks at a mass range of 400–600. The corresponding compounds were identified as bacillaene ([M + H]^+^ = 581.3582) by comparison with mass data obtained from *B. subtilis* XF-1 [28] (Figure 7C).

## 4. Discussion

*Bacillus* spp. have been widely studied as microbial biocontrol agents. As a useful microorganism in agriculture, *Bacillus* can affect plant growth and fruit-bearing by facilitating nutrient absorption and exerting various enzyme activities with antagonistic effects on pathogenic bacteria and fungi [29].

Cellulase enzymes degrade cell walls by cleaving the ß-1,4-D-glycosidic bonds that connect the glucose units containing cellulose and exert antifungal effects by destroying the fungal cell wall and cytoplasmic membrane [30]. Lysosomes and proteolytic enzymes capable of hydrolyzing polysaccharides adversely affect fungal growth and differentiation by dissolving or disturbing polymers in the cell wall of pathogenic fungi [30]. Lysozyme has been widely examined and shown to hydrolyze polymers belonging to the Gram (+) group present in the cell wall of bacteria; its ability to hydrolyze the cell wall of yeast and fungi was also recently reported [31,32]. The established potent activity of cellulase, lysozyme, and protease in TSA32-1 supports its association with the observed growth inhibition of various fungal phytopathogens in the present study (Figure 3). Furthermore, enzymatic lysis of the cell wall can increase chemical uptake into target cells [31]. In *Bacillus spp*. Vb1, Vb3, and Vb6, the high hydrolytic activity of enzymes, such as amylase, cellulase, and protease, is highly associated with antifungal activity against *F. oxysporum* pathogens [33]. The synergistic effect of co-production of cell wall hydrolytic enzymes and secondary metabolites useful for biocontrol may enhance the antagonistic effect on phytopathogenic fungi.

The antagonistic activity of TSA32-1 on phytopathogens revealed a broad capacity to inhibit the growth of fungal pathogens, which is a known characteristic of *Bacillus* species. This inhibitory ability of TSA32-1 may be related to the production of secondary metabolites. Interestingly, TSA32-1 showed antagonistic effects toward various plant pathogens that cause diseases such as root rot, head blight disease, bakanae disease, anthracnose, and damping-off disease. The positive impact of this antagonistic ability on soil-borne fungal pathogens of TSA32-1 is an important function as a microbial agent and increase its biocontrol potential.

Corn and pepper are important and representative food and vegetable crops. The most economically important diseases affecting these crops are blights disease, anthracnose disease, and damping-off disease [34]. Particularly, pathogenic *Fusarium* fungal species infect corn plants and reduce yields worldwide. The strain of *Pythium* and *Fusarium* such as *F. graminearum* and *F. oxysporum* are important pathogens of seedling diseases and interfere with seed germination and emergence, thus, affecting seedling development [35]. *B. velezensis* RC218 showed strong antagonist activity on seeds treated with *F. graminearum* [36]. In addition, Hyder et al. (2021) showed that the effects of *Bacillus* sp. on the germination rate of chili pepper seeds and its potential function as a biocontrol agent were evaluated by measuring the antagonistic effect and germination rate against the *Pythium* pathogen [37]. Analysis of the germination rate of corn and pepper seeds treated with TSA32-1 showed a higher germination rate than when each pathogen was treated alone (Figure 4). Application of TSA32-1 prior to pathogen exposure may induce tolerance in corn and pepper and activate plant defense responses against soil-borne pathogens, thereby limiting disease infection [38]. To complement field studies, pot experiments can allow for direct investigations without the influence of diffusing any environmental factors. Thus, seed or soil treatment pot experiments are performed to identify microbes as effective therapeutic agents against pathogens in agriculture [39]. Kim et al. (2017) showed that *B. amyloliquefaciens* JCK-12 significantly controlled *F. graminearum*-induced head blight in wheat under greenhouse and field conditions [40]. *B. subtilis* BBG100 was tested for its biocontrol potential in seed germination and pot experiments and found to reduce damping-off caused by *P. aphanidermatum* infection of tomato seedlings [41]. In addition, *B. subtilis* JA and D1/2 suppressed head blight and ear rot caused by *F. graminearum* in wheat, barley, and corn in the field [42]. *B. velezensis* TSA32-1 showed potential as a biocontrol agent by securing the ability to inhibit *F. graminearum* and *Pythium* on corn and pepper. Corn and pepper seeds treated with TSA32-1 were infected with soil-borne pathogens, and the sprout germination rate showed a tendency to increase when compared to the values of those infected with pathogens alone. Soil-borne pathogens like *F. graminaearum* and *Pythium* infection mainly occur in seeds, seedlings, and young roots, and it is known that it has a significant impact on the growth of seedlings, causing great economic loss and a ripple effect [37]. Against these soil-borne pathogens, TSA32-1 may contribute to the control effect that helps prevent disease in seeds. TSA32-1 exhibits biological control effects, including disease prevention, against several pathogens and crops, which could be a potential biocontrol agent.

Through genome analysis, we predicted the genes and gene clusters involved in the secondary metabolites producing TSA32-1. *Bacillus* spp. can produce multiple antimicrobials with a variety of chemical structures [43]. Lipopeptides are synthesized by NRPS. Generally, *Bacillus* strains produce the lipopeptides classified in the three families, iturin, surfactin, and fengycin, including plipastatin [44]. Among them, a cluster consisting of *srfAA*, *srfAB*, *srfAC*, and *srfAD* encoding the biosynthetic gene cluster of surfactin was detected in the TSA32-1 genome and had a size of 26.1 kb (Table 1). Surfactin is a multifunctional lipopeptide that exerts surfactant and signaling activity necessary for motility and biofilm development [45]. Plipastatin is a potent *Bacillus* antimicrobial lipopeptide with the potential for replacing conventional antifungal chemicals to control plant pathogens. In the genome of TSA32-1, we detected *ppsA, C, D, E* (37.6 kb) encoding the subunits of NRPS for plipastatin. The synthesized plipastatin may inhibit phospholipase A2, responsible for structuring pores in the fungal membrane and inducing the morphological change of membrane and cell wall of fungi [46]. In addition, the gene cluster *bae* of type I polyketide synthetases associated with the biosynthesis of bacillaene [47] were predicted in the TSA32-1 genome. The bae operon encodes an enzymatic macrocomplex that synthesizes bacillaene. Most *bae* genes appear to consist of large operons (>74 kb in *baeC-baeR*). Three *bae* operons were found in the genome of TSA32-1 with sizes of 43.3–71.7 kb. Accordingly, bacillaene of various sizes can be expressed and may improve antibacterial effects. Bacillaene is known as a broad-spectrum antibiotic that inhibits bacterial protein [48]. The gene cluster *ywfA-bacABCDE-ywfG-ywfH* encoding bacilysin synthesis was detected with a size of 7.9 kb. Bacilysin is known as an antibacterial substance that induces the dissolution of pathogen cell walls by inhibiting glucosamine-6-phosphate synthetase [49]. The *mycA-C* cluster also exists in the TSA32-1 genome. *myc* encodes mycosubtilin, which has an antifungal function. *B. subtilis* Z15, which produces mycosubtilin, applied to a cotton field confirmed its ability to inhibit wilt disease caused by *Verticillium dahlia* [50].

As described above, bacteria in the *Bacillus* genus produce a wide variety of antimicrobials. To confirm the genome background of secondary metabolites produced by TSA32-1, the culture medium was analyzed. The surfactin family is constituted by peptides of seven amino acids bound to the beta-hydroxyl fatty acid with 13–15 carbon atoms [51]. The fengycin family is represented by several isomers of fengycin, plipastatin, and maltacine; they consist of 10 amino acids connected to fatty acid with up to 18 carbon atoms [52]. The substances detected in TSA32-1 were similar in molecular weight to the two types of lipopeptides, and molecular ion peaks were detected as surfactin and plipastatin. The peaks show a molecular weight difference of 14 Da, suggesting that the length of the fatty acid chains varies within each group (CH_2_ = 14 Da). The signal detected in the chromatogram showed Rf value similar to the standard except for bacillaene. A peak similarly detected around an expected core peak was judged to have a water molecule omitted from the molecular formula of bacillaene. Bacillaene is an unstable secondary metabolite that is chemically sensitive to light, oxygen, and temperature [53]. In the 48 h culture of TSA32-1, it was difficult to detect this peak; the peak for bacillaene was detected in the 24 h culture.

Numerous studies and critical reviews have been conducted to determine the impact of *Bacillus* lipopeptides on plant pathogens [54]. *B. subtilis* pB2-L produced plipastatin (the fengycin family), which inhibits *F. oxysporum* mycelium growth [55]. *B. velezensis* GH1-13 showed high antagonistic activity toward various phytopathogenic fungi, including *F. fujikuroi* and *C. gloeosporioides*. Analysis of secondary metabolites revealed the presence of surfactin [56]. Gong et al. showed that *B. amyloliquefaciens* S76-3 produced plipastatin A and iturin A, which have clear antagonistic effects on *F. graminearum* [57]. *B. subtilis* BBG100 which is genetically engineered to overproduce mycosubtilin showed an antifungal effect against *Pythium* sp. by seed treatment on tomato [41]. Another classic example among other isolates is the plant-associated strain *B. amyloliquefaciens* FZB42 which produces antimicrobial peptides and secondary metabolites, including lipopeptides, polyketides, and bacteriocins while simultaneously producing substances that promote plant growth [58]. Many *Bacillus* strains produce more than one family of lipopeptides, and synergistic interactions may occur between different compounds to enhance biological control [59]. *B. velezensis* TSA32-1 also produces a variety of lipopeptides, such as surfactin, plipastatin, and bacilaene, with biological control effects. The investigation of these various lipopeptides and secondary metabolites provides scientific evidence of the effectiveness of TSA32-1 as a biocontrol agent.

## 5. Conclusions

*B. velezensis* TSA32-1 isolated from soil showed cellulase, lysozyme, and protease activities related to polymer degradation in the cell wall of plant pathogens and produced various lipopeptides such as surfactin, plipastatin, and bacillaene as secondary metabolites. *B. velezensis* TSA32-1 exerts antifungal action that destroys the structural integrity of fungal cells and inhibits the growth of phytopathogenic fungi such as *F. graminearum* and *P. ultimum*. Particularly, *B. velezensis* TSA32-1 reduced the occurrence of plant pathogens via antagonistic effects against *F. graminearum* and *P. ultimum*. In conclusion, *B. velezensis* TSA32-1 showed broad and effective antagonistic activity against various pathogenic fungi, suggesting its potential for use as a biocontrol microorganism in eco-friendly agriculture.

## Figures and Tables

**Table 1:** Secondary metabolite clusters identified in the genome of *Bacillus velezensis* TSA32-1.

Compound	Synthetase Type	Genes	Size (kb)	Bioactivity
Surfactin	NRPS	*srfAA*, *AB*, *AC*, *AD*	26.1	multiple
Bacilysin	NRPS	*ywfA*, *bacA*, *B*, *C*, *D*, *E*, *F*, *ywfH*	6.7	antibacterial
Plipastatin	NRPS	*ppsA*, *ppsC*, *ppsD*, *ppsE*	37.6	antifungal
Bacillaene	PKS-NRPS	*baeB*, *C*, *D*, *E*, *acpK*, *baeG*, *H*, *I*, *J*, *L*, *M*, *N*, *R*, *S* *baeC*, *L*, *M*, *ppsC*, *baeL*, *M* *baeC*, *G*, *I*, *L*, *M*	71.7 43.3 64.6	antibacterial
Mycosubtilin	PKS-NRPS	*mycA*, *B*, *C*	35.9	antifungal

PKS; Type I polyketide synthetases, NRPS; Non-ribosomal peptide synthetase.

## Data Availability

All sequences data were submitted to NCBI.
